# Molecular Basis of Neuronal and Microglial States in the Aging Brain and Impact on Cerebral Blood Vessels

**DOI:** 10.3390/ijms25084443

**Published:** 2024-04-18

**Authors:** Chihiro Maeda, Fuminori Tsuruta

**Affiliations:** 1 Master’s and Doctoral Program in Biology, Degree Programs in Life and Earth Sciences, Graduate School of Science and Technology, University of Tsukuba, 1-1-1 Tennodai, Tsukuba 305-8577, Ibaraki, Japan; s2320954@u.tsukuba.ac.jp; 2 Master’s and Doctoral Programs in Biology, Institute of Life and Environmental Sciences, University of Tsukuba, 1-1-1 Tennodai, Tsukuba 305-8577, Ibaraki, Japan; 3 Ph.D. Program in Human Biology, School of Integrative and Global Majors, University of Tsukuba, 1-1-1 Tennodai, Tsukuba 305-8577, Ibaraki, Japan; 4 Ph.D. Program in Humanics, School of Integrative and Global Majors, University of Tsukuba, 1-1-1 Tennodai, Tsukuba 305-8577, Ibaraki, Japan; 5 Master’s and Doctoral Program in Neuroscience, Graduate School of Comprehensive Human Sciences, University of Tsukuba, 1-1-1 Tennodai, Tsukuba 305-8577, Ibaraki, Japan

**Keywords:** aging, neuron, microglia, BBB, inflammation, neuronal nuclei

## Abstract

Brain aging causes a wide variety of changes at the molecular and cellular levels, leading to the decline of cognitive functions and increased vulnerability to neurodegenerative disorders. The research aimed at understanding the aging of the brain has made much progress in recent decades. Technological innovations such as single-cell RNA-sequencing (scRNA-seq), proteomic analyses, and spatial transcriptomic analyses have facilitated the research on the dynamic changes occurring within neurons, glia, and other cells along with their impacts on intercellular communication during aging. In this review, we introduce recent trends of how neurons and glia change during aging and discuss the impact on the brain microenvironment such as the blood-brain barrier (BBB).

## 1. Introduction

Aging is a phenomenon accompanied by complex processes that lead to a gradual deterioration in physiological functions and behavioral abilities [1]. In particular, as the brain governs numerous physiological functions and stands as the most intricate organ, the aging process in the brain gives rise to memory impairment, behavioral changes, cognitive decline, and disruption of immune systems [2]. Aging is also one of the risk factors for various neurodegenerative disorders, including Alzheimer’s disease (AD), Parkinson’s disease (PD), amyotrophic lateral sclerosis (ALS), and also for cerebrovascular disorders, such as cerebral ischemia and infarction [3]. It is well-known that the central nervous system (CNS) is composed of neurons and glia, including astrocytes, oligodendrocytes, and microglia. In addition, these neurons and glia, together with vascular endothelial cells, macrophages, and pericytes, constitute the microenvironment and maintain the BBB. The neurovascular unit (NVU) has been proposed as a conceptual framework for these microenvironments [4]. Vascular endothelial cells are adjacent to each other and form a barrier by junctional proteins such as Claudin-5, and pericytes localize around vascular endothelial cells. Astrocyte terminals also cover more than approximately 90% of capillaries and mediate communication between neurons and blood vessels [5,6]. Aging occurs in the various organs, and as the aging process advances, various biological, chemical, and physical functions undergo alterations in the molecules and cells of these organs. Brain aging in various mammalian species has several common features, including decreased synapses, regression of dendrites, and increased numbers of activated microglia and astrocytes [2]. Despite these identified features of brain aging, the precise molecular mechanisms observed in aging cells remain unclear. In this review, we first outline the general phenomenon of cellular senescence and introduce how neurons and microglia in the brain change during aging, comparing the differences with general cellular senescence. Then, the effects of these cellular changes on the BBB in the brain microenvironment will be introduced.

## 2. Cellular Senescence

Cellular senescence is a state of growth arrest in which cells lose their ability to proliferate despite proper growth conditions. It was first reported by Hayflick and Moorhead, who defined it in 1961 as the irreversible loss of replicative capacity of primary cells in culture [7]. Cellular senescence is caused by DNA damage, telomere dysfunction, and activation of oncogenes. It is known that a DNA lesion triggers the DNA damage response (DDR) pathway, which functions as a checkpoint and prevents cell cycle progression. DDR-related proteins accumulate at DNA damage sites and promote nuclear foci formation through post-translational modifications including histone H2AXphosphorylation [8] These nuclear foci represent each site of DNA damage and influence checkpoint function and cell cycle arrest until the damage sites are recovered [9]. One of the hallmarks seen in cellular senescence is the shortening of telomeres. The shortened telomeres are no longer protected by telomere regulatory factors and are similar to a situation of double-strand breaks (DSBs), resulting in the activation of the DDR pathway [10]. It has been also reported that the activation of oncogenes is another inducer of cellular senescence. Expression of oncogenes causes abnormalities in DNA replication and ultimately leads to cellular senescence by engaging the DDR pathway [11]. In addition, mitochondrial dysfunction is observed in senescent cells [12]. Mitochondria are cellular organelles that are the primary producers of intracellular energy via oxidative phosphorylation. Accumulation of dysfunctional mitochondria is also associated with increased oxidative stress in aging cells. Another feature observed in aging is autophagy dysfunction. Autophagy is a catabolic process that wraps misfolded proteins and damaged cell organelles in double-membrane vesicles, autophagosomes, which transport abnormal proteins and damaged organelles to lysosomes for degradation, serving as a quality control of proteins and organelles in cells [13]. However, with aging, autophagy-related proteins are downregulated, and the proteolytic function of lysosomes is reduced, impairing autophagy flux [14]. Additionally, aging cells are generally characterized by abnormalities in their nuclear structure. Expression of Lamin B1, one of the nuclear lamina components, has been reported be decreased during the aged stage [15]. Since Lamin B1 contributes to the attachment of heterochromatin to the nuclear membrane, the age-related decrease in Lamin B1 also causes the dissociation of heterochromatin from near the nuclear envelope. Lamin B1 preferentially binds to modifications associated with transcriptional repressions, such as histone H3 trimethylated at lysine 27 (H3K27me3) and lysine 9 (H3K9me3) [16]. Therefore, the age-related decrease in Lamin B1 is also accompanied by a decrease in H3K27me3 and H3K9me3, as well as a decrease in the heterochromatin content itself. This epigenetic change also affects the expression of the senescence-associated secretory phenotype (SASP) gene. The SASP is an important characteristic of senescent cells and releases numerous cytokines and chemokines associated with inflammation [17].

## 3. Neuronal Senescence

The intricate processing in the brain relies on the activity of neural circuits, constituting a higher order brain function. Therefore, the effects of neuronal aging on brain function are significant. It has been suggested that neuronal aging causes a decrease in the number of synapses, a decrease in gene expression related to synaptic plasticity, abnormal synaptic transmission in multiple brain regions, and a decrease in neurogenesis, leading to a decline in cognitive function [18]. Although replicative aging such as telomere loss is not observed in aged neurons, mitochondrial dysfunction, protein degradation abnormalities, SASP, and changes in nuclear morphology are common features in aged neurons as well as in other aged cells. Since the brain requires enormous amounts of energy for regulating synaptic connectivity, mitochondrial dysfunction with aging is prominent in neurons due to a lack of energy. In addition, they are more sensitive to the accumulation of oxidative damage than cells undergoing mitosis. A mathematical model has been proposed in which mitochondria damaged by aging tend to accumulate [19]. Indeed, small, rounded, and fragmented mitochondria have been observed in dopaminergic neurons in aged mice. These studies suggest that the accumulation of damaged mitochondria is associated with a decrease in dopaminergic neurons in an age-dependent manner [20]. Therefore, it seems that mitochondrial dysfunction is one of the major causes of age-related neuronal disorders. Moreover, mitochondria are equipped with a protein stress response mechanism known as the mitochondrial unfolded protein response. This cellular response to protein stress is observed in mitochondria during aging. In the neural stem cells of the dentate gyrus, mitochondrial protein folding stress has been demonstrated to increase with age [21]. Additionally, increased mitochondrial protein folding stress is found to compromise neural stem cell maintenance and reduce neurogenesis, resulting in neural hyperactivity and cognitive dysfunction. However, SIRT7 has been identified as a protective factor for neural stem cells, as it suppresses mitochondrial protein folding stress [21]. Therefore, the overexpression of SIRT7 has the potential to augment neurogenesis and improve cognitive function in aging mice.

It has also been shown that the expression of autophagy-related protein 5 (ATG5) and ATG7, key proteins in autophagosome formation, is decreased in aging human brain tissue [22]. Furthermore, the accumulation levels of Atg5 and phosphatidylinositol 3-phosphate (PI3P) recognized by the GFP-2xFYVE domain, which is a probe of PI3P, were gradually decreased in the brain of *Drosophila* [23]. Conversely, the levels of the lipidation form of Atg8a/microtubule-associated proteins 1A/1B light chain 3B, which is the autophagic membrane-bound form of Atg8a (Atg8a-II), were increased. These findings imply that autophagy dysfunction in the aging brain manifests at two distinct levels. Given that the FYVE domain binds to PI3P, the abundance of the GFP-2xFYVE domain correlates with phosphatidylinositol-3 kinase (PI3K)/Vacuolar protein sorting-associated protein 34 (Vps34) activity. This decline in Atg5 and the 2xGFP-FYVE domains signifies an impairment at the phagocytosis/autophagosome formation stage. Atg8a-II levels are gradually increased with age despite a reduction in autophagosome formation and impair autolysosome functions. This suggests that dysfunction also arises after autophagosome formation, either during the fusion of the structure with lysosomes or the enzymatic digestion of autolysosome contents. Furthermore, the inhibition of autophagy increases the number of neurons with elevated SA-β-gal activity [24], suggesting another possibility that dysfunctional autophagy contributes to neuronal aging.

**Figure 1:**
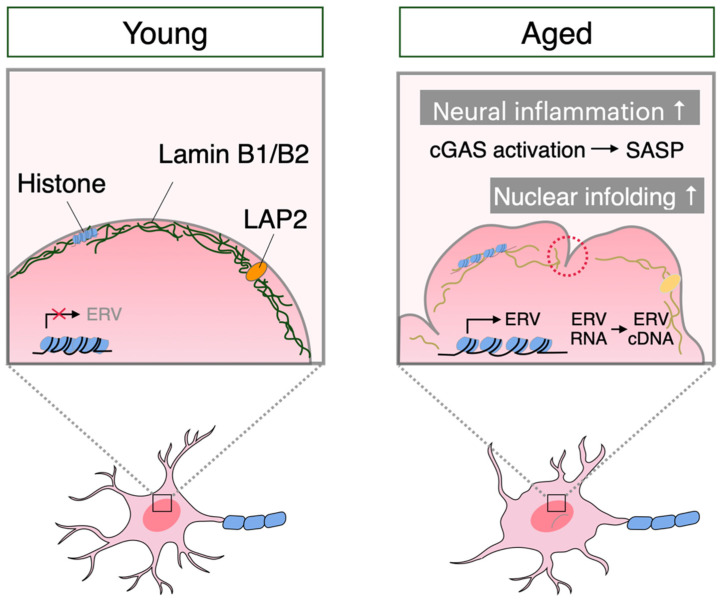
Changes in neuronal nuclei during aging. During the aging process, neurons show abnormal nuclear structure due to decreased expression of nuclear envelope proteins, such as Lamin B1, Lamin B2, and LAP2β. This abnormality in the nuclear structure triggers the activation of endogenous ERVs and activates the cGAS-STING pathway, leading to increased proinflammatory cytokine secretion. In addition, nuclear infolding increases with aging. The nuclear structure of neurons plays an important role in the regulation of gene expression and chromatin structure. The precise analyses of molecular mechanisms that underlie the abnormalities of the neuronal nuclear envelope with aging is a key to understand the physiological and pathological phenomenon of brain aging.

Abnormalities in the nuclear structure are also common in neuronal senescence (Figure 1). It has been reported that aged primate frontal lobe neurons also show decreased transcript levels of LMNB1 and LMNB2, the constituent factor of the nuclear lamina, and LAP2β, a heterochromatin-related inner membrane of the nuclear envelope [25]. Along with this reduction in subnuclear heterochromatin, the integrity of the nuclear lamina was also found to be impaired. This neuron-specific change in nuclear architecture leads to the activation of endogenous retrovirus (ERV) retrotransposons, which in turn triggers the activation of the cGAS-mediated innate immune response downstream, inducing an increase in proinflammatory cytokines. In addition, knockdown of Lamin B1 and Lamin B2 using siRNA in human embryonic stem cell (hESC)-derived neurons resulted in the loss of heterochromatin and activation of the cGAS pathway via ERV retrotransposons, as well as increased Aβ accumulation and gene expression involved in senescence and inflammation [25]. This also suggests that the starting point of the neuronal senescence cascade is the loss of LaminB1 and Lamin B2. Neurons also undergo complex changes in nuclear morphology following external environmental stimuli, which play an important role in the regulation of gene expression. It has been shown that cell morphology influences the shape and size of the nucleus, which may affect cell function. Recent studies have reported that the size and shape of neuronal nuclei, which change with age and disease, also vary from region to region of the brain [26]. Twenty-four-month-old aging mice show that the size of neuronal nuclei in the neocortex and striatum decreases compared to those in young mice. In addition, the roundness of nuclei in striatal neurons significantly decreased, and the size of nuclei in hippocampal CA3 pyramidal neurons increased with age. Furthermore, neural activity induced by exposure to a novel environment caused a reduction in the size of nuclei and an increase in the number of nuclei with infolding. These results also indicate that neuronal activity induced by external stimuli alters nuclear morphology, which may be crucial for regulating gene expression and chromatin structure. It has also been reported that this nuclear infolding is a reversible process [27]. In the nuclei of young neurons, morphological changes of the nuclear periphery were observed within 10 min after stimulation, and the nuclei returned to a spherical shape when the stimulus was removed, whereas in the nuclei of aged neurons, the dynamic behavior of the nuclei was reduced. Atomic force microscopy analysis revealed that nuclei stiffen in aging neurons, suggesting that nuclear stiffness is related to the loss of dynamics of nuclear shape with aging. 

Furthermore, nuclear infolding has been reported to be a phenomenon also seen in neurodegeneration: in neural progenitors and hippocampal neurons in PD patients with PD-associated leucine-rich repeat kinase 2 (LRRK2) G2019S mutations and in the midbrain of transgenic mice ectopically expressing the PD-associated LRRK2 R1441C mutation, the nuclei in dopaminergic neurons show irregular shapes [28]. Multiple missense mutations in LRRK2 are known to be associated with familial late-onset PD, and the loss of LRRK2 induces age-related dendritic atrophy, cell body enlargement, and nuclear infolding. Furthermore, increased DNA damage and abnormal histone methylation were detected in aging *Lrrk2*^−/−^ striatal neurons, as well as changes in the molecular bases that underlie activation of the neuronal circuit, genomic stability, and protein quality control, suggesting that LRRK2 mutations promote the aging process, thereby causing neural degradation of cellular structures by accelerating the aging process. A high incidence of nuclear infolding is also known in hereditary frontotemporal dementia (FTD), which is caused by tau mislocalization [29]. In FTD, the neuronal microtubule-associated protein tau (MAPT) is hyperphosphorylated and relocalized to the cell bodies and dendrites of cortical neurons. The abnormal distribution of tau in the soma causes aberrant microtubule movement in neurons and microtubule invasion into the nucleus, resulting in nuclear membrane deformation and nuclear infolding.

In recent years, research on abnormalities in the neuronal nuclear structure has advanced significantly, not only in neurodegenerative diseases but also in normal aging. Moving forward, it will be important to deepen our understanding of the upstream mechanisms leading to abnormalities in neuronal nuclei and their downstream phenomena.

## 4. Microglial Senescence

Microglia are immune-responsive cells residing in the brain and play a vital role in brain homeostasis from embryonic development to old age [30,31]. Microglia originate from yolk sac progenitors during embryonic development, migrate into the brain primordium, proliferate during pre- and postnatal stages, then colonize the CNS and self-renew throughout life [32,33,34]. Besides immunity, microglia perform various essential functions, including synaptic pruning [35,36], phagocytosis of apoptotic neurons [37], neurogenesis [38,39], synapse formation [40], and regulation of the BBB [41].

Normally, adult microglia have branched projections and cell bodies, but they enter an activated state and change morphology in response to stimuli, aging, or progressive CNS pathology. Recent studies have reported heterogeneous populations of microglia in the brain across organisms of different lifespans, sexes, and species [42,43]. Transcriptome analysis showed that microglia in female mice show an advancing aging process in each stage, while microglia in male mice switch abruptly to an aging phenotype at 12 months. scRNAseq analyses have revealed that gene signatures of interferon-responsive microglia in the aging brain are changed [44]. Microglial populations found in the aging brain are characterized by increasing gene expressions, such as *Lgals3*, cystatin F (*Cst7*), chemokines *Ccl4* and *Ccl3*, cytokine interleukin 1β (*Il1b*), interferon-induced transmembrane protein 3 *(Ifitm3*), receptor transporter protein 4 (*Rtp4*), and interferon response genes, 2′-5′ oligoadenylate synthase-like 2 (*Oasl2*). This suggests that the subpopulations of microglia regulate age-related inflammation in the brain. Additionally, age-dependent white matter-associated microglia (WAM) have recently been identified [45]. WAM share some disease-associated microglia (DAM) gene signatures and are also characterized by the gene involved in phagocytosis and lipid metabolism. WAM show the downregulation of homeostatic genes such as purinergic receptors *P2ry12* and *P2ry13,* and homeostatic genes such as *Csfr1r*, *Cx3cr1*, *Hexb*, and *Tmem119*. Conversely, WAM exhibit the upregulation of DAM-related genes such as *ApoE*, *Bm2*, *Cst7*, *Cd63*, *Clec7a*, *Ctsb*, *Ctss*, *Ctsz*, *Lyz2*, *H2-D1,* and *H2-K1*. Furthermore, WAM appearance depends on triggering receptor expressed on myeloid cells 2 (TREM2) signaling in an age-dependent manner. Moreover, microglia that accumulate lipid droplets are suggested to be defined as lipid droplet-accumulating microglia (LDAM) [46]. Such microglial populations are known to exhibit a phagocytosis deficit, reactive oxygen species (ROS) production, and inflammatory cytokine secretion. CRISPR-Cas9 screening has revealed that various genes including *SLC33A1*, *SNX17*, *VPS35*, *CLN3*, *NPC2*, and *GRN* are associated with microglial lipid droplet formation. Thus, LDAM seem to be a pivotal subpopulation that underlies aged brain homeostasis and neurodegeneration.

Using bulk RNA-seq, the mouse brain transcriptome was examined at various stages of adulthood, and age-dependent microglia (ADEM) genes that are upregulated or downregulated in old age were identified [42]. ADEM genes which show a positive correlation with aging include the interferon signaling (*Cxc16*, *Gas6, Ifitm2*, *Ifi204*, *Tgtp2*, *Xaf1*), antigen presentation (*H2-D1*, *H2-Q7*, *H2-K1Cd74*, *Tap1*), lipid metabolism (*Apod*, *Lpl*, *Spp1*), immune response (*Ccl6*, *Ccl12*, *Il1rn*, *Tnf*), phagocytosis (*Axl*, *Cst7*, *Fcgr3*, *Spp1*), and oxidative stress response genes *(Cybb*, *Hp*). ADEM genes that show a negative correlation with age include microglial marker genes (*Fcrls*, *Il4ra*), chemokine signaling (*Il4ra*, *Socs3*, *Tlr7*), ER-associated protein degradation (ERAD)-related genes (*Hspa13*), and iron metabolism genes (*Fth*, *Hfe*). Gene ontology analyses have revealed that ADEM-related genes are involved in immune cell migration, cytokine and chemokine signaling, and immune response pathways. Importantly, 21 ADEM genes are overlapped with DAM genes. Of these genes, *Axl*, *Cd74*, *Cst7*, *Cybb*, *Fth1*, *Spp1*, *Lpl*, and *H2-D1* are observed in both physiological and pathophysiological processes in the aging brain, indicating that the ADEM gene is highly correlated with the etiology of neurodegeneration. In addition, CEBPβ and MEF2C, which regulate microglial homeostasis and reactivity in the aging brain, are potential key mediators that link ADEM genes to characteristic subpopulations. Thus, microglia show dramatic changes with aging, including altered gene expression, loss of molecular signatures maintaining homeostasis, increased production of inflammatory cytokines, increased generation of ROS, and accumulation of dysfunctional lysosomes indicating impaired phagocytic function.

Concerning the age-related loss of phagocytic capacity, the normal B cell receptor CD22 has been identified as a negative regulator of phagocytosis [47]. CD22 has been upregulated by aging and regulates the anti-phagocytic effect of α2,6-linked sialic acid. CD22 suppression promotes clearance of myelin remnants and neurodegenerative-related factors, including amyloid β-oligomers and α-synuclein fibers in vivo. Persistent inhibition of CD22 partially influences age- and disease-related microglial gene expression profiles, such as reduced inflammatory gene expression and increased markers of neuronal activation, improving cognitive function in aging. Recent studies have reported that microglial CD22 is upregulated at postnatal day 7 (P7). Because microglia play an important role in eliminating apoptotic oligodendrocytes at the early-postnatal stages, it is likely that upregulation of CD22 serves as a negative feedback machinery to alleviative excessive phagocytosis, suggesting that the involvement of CD22 acts as a negative regulator of phagocytosis in aging microglia as well.

TREM2 also plays a major role in the regulation of microglial function [48]. TREM2 recognizes bacterial lipopolysaccharides, sulfated glycosaminoglycans, and phospholipids, and upon stimulation, associates with the adapter molecule TYROBP/DAP2. TREM2 mutations are known to be associated with age-related neurodegenerative disorders. Although the role of TREM2 that underlies the aging process in the brain remains unclear, the analyses of aged TREM2 knockout (KO) mice have shown that TREM2 mutations alter gene expression profiles in microglia, such as microglial markers (*Aif1*, *Tmem119*, *Cd68*), oxidative stress markers (*Cyba*, *Cybb*, *Inos*), and complement components (*C1qa*, *C1qb*, *C1qc*, *C3*, *C4b*, *Itgam/Cd11b/CR3A*, *Itgb2/Cd18*). These results indicate that a loss of TREM2 attenuates microglial activation and reduced oxidative stress in the aged brains of TREM2 KO mice. Dysfunction, such as diminished microglial phagocytosis, leads to the accumulation of abnormal myelin debris, such as lipid droplets and lipofuscin granules, in microglia. This debris has also been observed to exacerbate microglial dysfunction and myelin degeneration, influencing brain aging.

The cGAS-STING pathway plays an important role in regulating neuroinflammation and aging in the CNS [49]. Indeed, recent studies have also revealed that the cGAS-STING pathway is involved in the inflammation of aging microglia [50]. Activation of STING exhibits reactive microglial transcriptional status involved in neurodegeneration and cognitive dysfunction. Importantly, cytoplasmic DNA released from mitochondria induces activation of cGAS in aging microglia. This activation of GAS-STING signaling is essential for the type I IFN response in the aging-associated cellular phenotype in microglia, leading to neuronal damage and cognitive dysfunction. 

It has long been known that aging microglia have increased responsiveness to stimuli and decreased function. On the other hand, recent technology has revealed that microglia have very diverse and complex phenotypes even in the aging brain. However, with such a rich diversity of microglia, it is not yet fully understood how microglia communicate with each other or between microglia and other cells. Thus, this intriguing question will need to be further studied in the future to understand these mechanisms precisely.

## 5. Aging of the Blood-Brain Barrier

The BBB maintains brain homeostasis by regulating the exchange of substances between the brain parenchyma and blood. The BBB forms a specific organization through interactions between brain vessel endothelial cells that express intercellular tight junctions, and these complex tight junctions seal the intercellular space and form a barrier [51]. In addition, specific transporter proteins in endothelial cells regulate molecules entering and leaving the brain. Tight junction proteins of brain endothelial cells include the Occludin and Claudin family proteins, Zonula occludens (ZO1, ZO2, ZO3), membrane-bound guanylate kinase (MAGUK) protein families, Cadherin, catenin, and PECAM-1. These proteins regulate the proper formation and stabilization of cell–cell interactions in endothelial cells [52]. Claudin-5, in particular, is a major component of tight junctions and contributes to reducing intercellular ion exchange, thereby helping to narrow the intercellular gap [53]. Moreover, transforming growth factor-β (TGF-β) has been proposed to regulate cellular adhesion between vascular endothelial cells and pericytes. TGF-β signaling in pericytes promotes gene expression of extracellular matrix proteins, such as fibronectin, and contributes to the formation of basement membranes [54]. Recent studies have suggested that pericytes play a major role in maintaining BBB integrity through the regulation of gene expression profiles in endothelial cells. In addition, pericytes control the polarization of astrocyte end-foot processes [55,56]. The end feet of astrocytes overlap each other and are in direct contact with the endothelial basement membrane, which wraps around the vasculature [5]. The channel clustering of the water channel aquaporin 4 (AQP4) and the potassium channel Kir4.1 reside in the endfeet [57]. These clusterings regulate the exchange of water and solutes between the blood and the brain. This astrocyte endfeet do not directly contribute significantly as a physical barrier, but soluble factors derived from astrocytes such as glial cell line-derived neurotrophic factor (GDNF), basic fibroblast growth factor (bFGF), FGF-2, TGFβ, IL-6, and angiopoietin 1 (ANG1) mediate BBB integrity in a context-dependent manner. Indeed, transplantation of cultured astrocytes into leaking vessels restores leaking vessels by tightening endothelial cells indicating intercellular communication in the neighborhood of vessels [58]. The destruction of the BBB is a common phenomenon observed in normal aging. The aging-associated destruction of the BBB includes degradation and shrinkage of endothelial cells, followed by decreasing expression of tight junction proteins, as well as pericyte degeneration and decreased pericyte coverage [59,60], changes in the basement membrane [61], and detachment of astrocyte ends from the vascular basement membrane. When blood exudes as a result of the destruction of the BBB, the perivascular cells undergo various changes.

**Figure 2:**
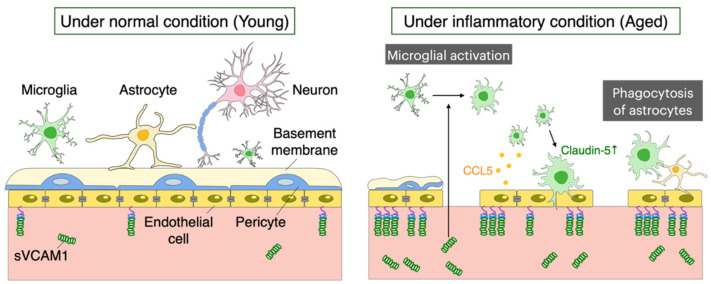
Changes in BBB structure with aging. Disruption of the BBB with aging results in loss of endothelial cells and shrinkage, downregulated expression of tight junction proteins, decreased pericyte coverage, basement membrane changes, and astrocyte detachment. VCAM1 is shed from aging vascular endothelial cells and induces microglial activation. Sequentially, microglia change to a phagocytic state with persistent inflammation, removing fragments of astrocyte foot processes, causing leakage through the BBB and contributing to reduced BBB integrity. sVCAM1; soluble VCAM1.

Recently, it has been shown that blood proteins also affect microglial transcription [62]. Blood proteins have been shown to regulate microglial gene expression, including ROS-related genes (*Hmox1*, *Romo1*, *Gpx1*) and DAM-related genes (*Cst7*, *Spp1*), indicating that blood proteins induce a wide range of microglial transcriptional changes. Furthermore, the deletion of the blood coagulation factor, fibrinogen, nearly reversed the blood-induced microglial neurodegeneration signature, indicating that blood proteins have a significant effect on microglial activation. It has also been reported that vascular cell adhesion molecule 1 (VCAM1) shed from aging vascular endothelial cells also activates microglia [63] (Figure 2). VCAM1 is a protein that facilitates the interaction of blood vessels with immune cells and is found in brain endothelial cells in aging mice, which exhibit a similar inflammatory transcriptional profile. At the same time, levels of the effluxed soluble form of VCAM1 were markedly elevated in the blood during aging. In contrast, systemic administration of anti-VCAM1 antibodies and genetic disruption of VCAM1 in brain endothelial cells counteracted the detrimental effects of aged plasma on the young brain and reversed aspects of aging, including microglial changes in the aged mice brains.

The full mechanism by which the NVU is involved in the destruction and repair of the BBB during aging remains unclear. During inflammation, microglia contribute to both the protection against and loss of BBB integrity. Upon the onset of inflammation, microglia first respond to the released CCL5 from endothelial cells, accumulating around cerebral blood vessels before any changes in BBB permeability occur (Figure 2). This process gives rise to the expression of Claudin-5 in microglia, infiltrating through the neurovascular unit, and forming tight junctions in contact with endothelial cells. On the other hand, if this inflammatory state persists, microglia change to a phagocytic state and eliminate fragments of astrocyte foot processes, causing leakage through the BBB and reducing BBB integrity. The actions of microglia that control BBB integrity are opposing over time and through signaling pathways, suggesting a dual role in the maintenance of BBB integrity [41]. Because brain aging induces inflammation, understanding the mechanism linking microglia and vascular endothelial cells during inflammation may also provide insights into comprehending the process of brain aging. In addition, RNA sequencing of microglia and astrocytes before and after changes in BBB permeability in normally aged mice at 12 months of age revealed that microglia show significant changes in protein phosphorylation and negative regulation of phagocytic vesicles, whereas astrocytes show no significant changes in the restoration of BBB function due to enzymes and peptidase inhibitory activity [64].

The loss of BBB integrity is a phenomenon observed in the physiological aging state, but BBB disruption causes the phenomenon to be exacerbated in neurodegenerative diseases. Disruption of the BBB is a major factor in AD, PD, ALS, multiple sclerosis (MS), and Huntington’s disease (HD). However, the precise mechanisms that cause these neurodegenerative disorders have not been elucidated. Furthermore, it remains controversial whether the dysfunction of the BBB in a disease is a causative agent or a consequence of the disease.

## 6. Conclusions

Recent technological advances provide experimental evidence of cellular-level changes in neurons, glia, and vascular endothelial cells. Particularly, certain studies suggest that the abnormal nuclear structure in neurons serves as the initiation point for neuronal aging and may play a pivotal role in elucidating the aging mechanism. Neurons and glia, in conjunction with vascular endothelial cells, and pericytes, collectively form the microenvironment responsible for maintaining the BBB. Determining the chronological sequence of aging events within these microenvironments presents a significant challenge. This constitutes a crucial endeavor in identifying potential therapeutic mechanisms aimed at preserving or enhancing BBB integrity. Moreover, addressing fundamental questions such as the primary targets for aging therapy within the brain and the optimal implementation of aging therapy will foster novel and innovative approaches to aging treatment in the future.

## Abbreviations

BBB, blood-brain barrier; AD, Alzheimer’s disease; PD, Parkinson’s disease; ALS, amyotrophic lateral sclerosis; MS, multiple sclerosis; HD, Huntington’s disease; FTD, frontotemporal dementia; CNS, central nervous system; NVU, neurovascular unit; DDR, DNA damage response; DSBs, double-strand breaks; SASP, senescence-associated secretory phenotype; PI3P, phosphatidylinositol 3-phosphate; ERV, endogenous retrovirus; hESC, human embryonic stem cell; WAM, white matter-associated microglia; DAM, disease-associated microglia; LDAM, lipid droplet-accumulating microglia; ADEM, age-dependent microglia; ROS, reactive oxygen species; ERAD, ER-associated protein degradation.
